# Downregulation of β1,4-galactosyltransferase 5 improves insulin resistance by promoting adipocyte commitment and reducing inflammation

**DOI:** 10.1038/s41419-017-0239-5

**Published:** 2018-02-07

**Authors:** Shu-Fen Li, Cui-Song Zhu, Yu-Meng Wang, Xin-Xin Xie, Liu-Ling Xiao, Zhi-Chun Zhang, Qi-Qun Tang, Xi Li

**Affiliations:** 1 Key Laboratory of Metabolic Molecular Medicine, the Ministry of Education, Department of Biochemistry and Molecular Biology, School of Basic Medical Sciences, Fudan University Shanghai Medical College, Shanghai, 200032 China; 2 Biology Science Institutes, Chongqing Medical University, Chongqing, 400032 China; 3 State Key Laboratory of Medical Genomics and Shanghai Institute of Hematology, Ruijin Hospital, Shanghai Jiao Tong University School of Medicine, Shanghai, 200025 China

## Abstract

Protein glycosylation is an important post-translational modification. Aberrant glycosylation has been implicated in many diseases because of associated changes in protein distribution and biological function. We showed that the expression of β1, 4-galactosyltransferase 5 (B4GalT5) was positively correlated with diabetes and obesity. In vivo, B4GalT5 knockdown in subcutaneous adipose tissue alleviated insulin resistance and adipose tissue inflammation, and increased adipogenesis in high-fat diet (HFD)-fed mice and ob/ob mice. Downregulation of B4GalT5 in preadipocyte cells induced commitment to the adipocyte lineage in the absence of bone morphogenetic protein (BMP) 2/4 treatment, which is typically essential for adipogenic commitment. RNAi silencing experiments showed B4GalT5 knockdown activated Smad and p38 MPAK signaling pathways through both type 1A and 2 BMP receptors. Remarkably, B4GalT5 knockdown decreased BMPRIA glycosylation but increased BMPRIA stability and cellular location, thus leading to redistribution of BMPRIA and activation of the BMP signaling pathway. Meanwhile, downregulation of B4GalT5 decreased the infiltration of macrophages and the markers of M1 macrophages in subcutaneous adipose tissue of HFD mice and *ob/ob* mice. In bone marrow-derived macrophages (BMDMs) and RAW264.7cells, B4GalT5 knockdown also repressed the markers of M1 by reducing NFκB and JNK signaling. These results demonstrated B4GalT5 downregulation improved insulin resistance by promoting adipogenic commitment and decreasing M1 macrophage infiltration.

## Introduction

Obesity, characterized by the expansion of white adipose tissue (WAT), is a complex disorder and a major risk factor for metabolic diseases, such as insulin resistance, type 2 diabetes (T2D), hypertension, and atherosclerosis^1,2^. Elucidating the mechanisms underlying obesity is important for effective treatment of associated diseases. WAT expands by hyperplasia and hypertrophy. Adipocytes development occurs in two progressive stages: the commitment of mesenchymal stem cells (MSC) to preadipocytes and the terminal differentiation of preadipocytes^3^. The C3H10T1/2 cells, derived from C3H mouse embryos, are MSCs, which require BMP signaling to induce commitment to adipocyte lineage cells^4,5^. It is one of the faithful in vitro models for long-term genetic studies of the adipocyte developmental program^6,7^.

Clinical studies have demonstrated that obese individuals are also divided into two types: metabolically healthy obese (MHO) and metabolically abnormal obese (MAO). According to cross-sectional studies, MHO individuals had smaller-sized adipocytes than MAO patients^8^. Adipocyte size is an important determinant of adipokine secretion, large adipocyte size is positively correlates with the secretion of macrophage inflammatory protein-1β, interleukin (IL)-6, monocyte chemoattractant protein-1 (MCP-1), and negatively correlated with IL-10 secretion^9^. Our group also reported that large adipocytes activated CD4^+^T cells via upregulating interferon (IFN)-γ and promoted adipose tissue inflammation^10^. These findings suggested that adipocytes hypertrophy was associated with the development of metabolic disorders and small-size adipocytes were beneficial to maintain adipose tissue homeostasis.

As noted, ample evidences demonstrated that obesity was a chronic low-grade inflammatory state^11,12^. Obesity-induced the changes of macrophages and adipocytes leaded to chronic inflammation and insulin resistance^13^. Two major macrophage phenotypes have been described in obesity: classically activated Macrophage or M1, which triggers a proinflammatory effect, and alternatively activated Macrophage or M2, which promotes anti-inflammatory effect. In lean, the adipose tissue macrophages (ATMs) are mainly M2 macrophage exhibiting an anti-inflammatory effect. With the development of obesity, adipocytes can release proinflammatory mediators, such as CC chemokine ligand (CCL)-2 (also known as MCP-1), tumor necrosis factor (TNF)-α, free fatty acids (FFAs), which recruit M1 macrophage. In turn, M1 cells express Itgax and high levels of iNOS, TNF-α, and IL-6, which impede insulin signaling in adipocytes and promote obesity-associated inflammation and insulin resistance^14^.

Protein glycosylation is an important post-translational modification that regulates various biological functions^15^. Glycans have well-documented roles in protein folding, endocytosis, trafficking, and function^16–19^, and glycan structures are largely determined by the expression pattern and substrate specificities of glycosyltransferases. Thus, the glycosyltransferase enzyme family is an attractive target for genetic investigation of the function of protein glycosylation^20^. The β1, 4-galactosyltransferase (B4GalT) enzyme family transfers galactose (Gal) from uridine diphosphate galactose to *N*-acetylglucosamine (GlcNAc)-terminated oligosaccharides to form *N*-acetyllactosamine^21^. The lack or alterations in the activity of some B4GalT subfamily members have been associated with various diseases, especially cancer^22–24^. However, the biological functions of B4GalTs in obesity and diabetes are poorly understood.

Recent research investigating the *N*-glycosylation profile of undifferentiated and adipogenically differentiated human bone marrow MSC show that linear poly-*N*-acetyllactosamines (poly-LacNAc) are one of the main glycan changes found in undifferentiated MSCs^25,26^. Poly-LacNAc epitopes interact with lectins such as galectins, which play important roles in obesity and insulin resistance^27,28^. Since B4GalTs are the main enzymes involved in biosynthesis of poly-LacNAc, they may be involved in adipocyte development and obesity. Indeed, recent studies have showed that B4GalT5 was upregulated upon TNF-alpha-induced insulin resistance in adipocytes^29^. This suggests that B4GalT5 may be involved in the regulation of obesity and insulin resistance.

Here, we demonstrated that the expression of B4GalT5 was upregulated during obesity and diabetes both in human and mice. Downregulation of B4GalT5 expression in subcutaneous adipose tissue alleviated insulin resistance, inflammation, and improved metabolic status of high-fat diet (HFD)-induced obesity and *ob/ob* mice by promoting adipogenic commitment and reducing macrophage inflammation in adipose tissue.

## Results

### B4GalT5 expression was increased in type 2 diabetes and obesity

**Fig. 1:**
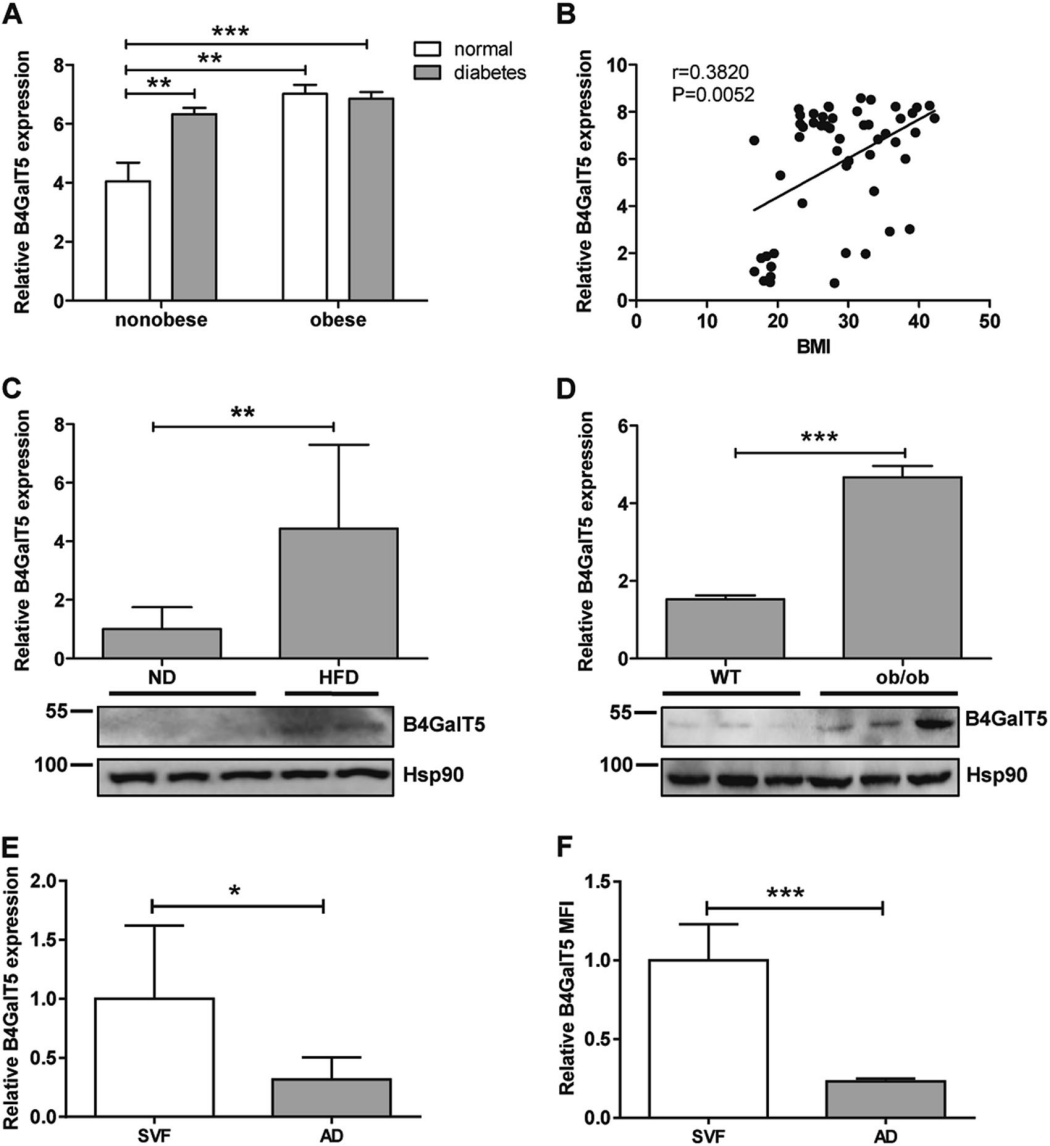
B4GalT5 expression is positively correlated with type 2 diabetes and obesity. **a** B4GalT5 expression in subcutaneous adipose tissue from subjects with neither obesity nor diabetes (*n* = 12) vs. those without obesity but with diabetes (*n* = 9); and from subjects with obesity but without diabetes (*n* = 6) vs. with both obesity and diabetes (*n* = 13). **b** Correlation analysis between BMI and B4GalT5 mRNA levels in subcutaneous adipose tissue of human (*n* = 52). **c**–**d** qPCR analysis of B4GalT5 mRNA expression and western blotting detect B4GalT5 protein level in inguinal (subcutaneous) adipose tissue of C57BL/6 J mice fed with ND or HFD for 12 weeks (*n* = 14), and *ob/ob* mice (*n* = 8) and their wild-type(WT) littermates (*n* = 8). **e** B4GalT5 mRNA expression in stromal vascular fraction (SVF) or mature adipocytes fraction (AD) isolated from inguinal adipose tissue of C57BL/6 J mice fed with HFD for 12 weeks (*n* = 6). **f** The relative mean fluorescence intensity (MFI) by flow cytometric analysis of SVF and AD for B4GalT5 expression in inguinal adipose tissue of C57BL/6 J mice fed with HFD for 12 weeks (*n* = 6). Statistical analysis was performed by collating the results from multiple samples. *P < 0.05, **P < 0.01, ***P < 0.001

To address the potential role of B4GalT5 in obesity and T2D, we first detected B4GalT5 expression in human subcutaneous adipose tissue. We found that B4GalT5 expression was significantly increased in T2D patients (Fig. 1a). And the expression of B4GalT5 was positively correlated to adiposity, i.e., body mass index (BMI) (Fig. 1a, b). Then we detected B4GalT5 expression in subcutaneous adipose tissue of HFD mice and *ob/ob* mice, which were hyperglycemic and exhibited insulin resistance. B4GalT5 was significantly increased in these mice at the mRNA and protein level (Fig. 1c, d). In HFD mice, the vast majority of B4GalT5 was expressed in the stromal vascular fraction (SVF), which contains preadipocytes in various stages and multiple types of immune cells including high ratio of macrophages, with a relatively small proportion expressed in the mature adipocyte fraction (Fig.1e, f). These data demonstrated that B4GalT5 expression was positively correlated with diabetes and obesity and indicated the potential roles of B4GalT5 in preadipocytes and macrophages.

### B4GalT5 knockdown in subcutaneous adipose tissue alleviated systemic insulin resistance and adipose tissue inflammation

**Fig. 2:**
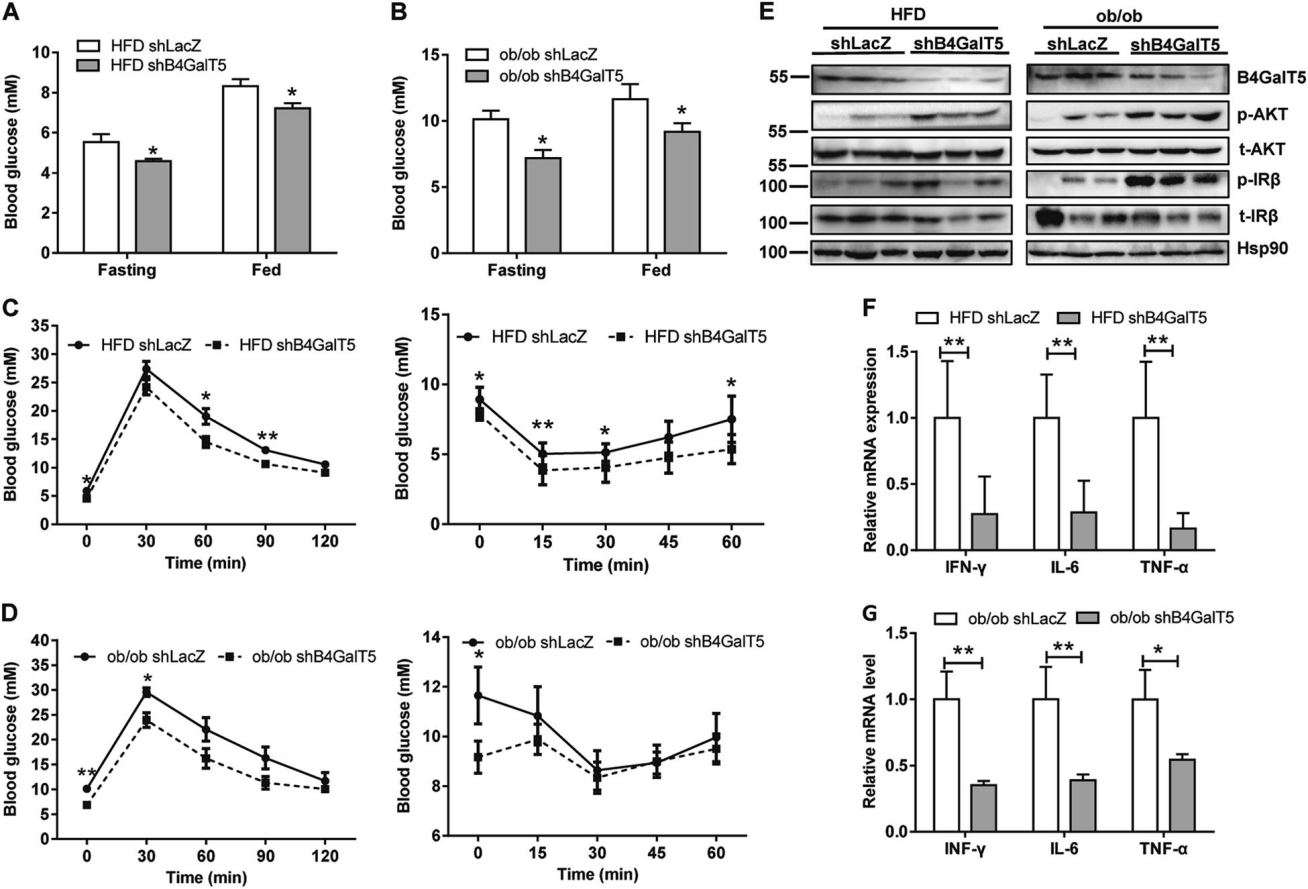
B4GalT5 knockdown in subcutaneous adipose tissue prevents obesity-induced insulin resistance and adipose inflammation. Six-week-old C57BL/6 J mice and 4-week-old *ob/ob* mice were injected with adenovirus expressing B4GalT5 or control LacZ shRNA twice a week s.c. adjacent to both sides of the inguinal fat pads for 6 weeks. WT groups of mice were fed with HFD, *ob/ob* mice were fed with ND. **a–b** Blood glucose was measured under fasting and fed conditions (*n* = 6/group). **c**–**d** GTT and ITT assays were conducted in HFD mice (*n* = 6/group) and *ob/ob* mice (*n* = 6/group). **e** Western blotting analysis of the insulin-signaling pathways in HFD-fed mice and *ob/ob* mice treated with B4GalT5 or LacZ shRNA adenovirus. These mice were killed after insulin stimulation (0.75 mU/g body weight) for 5 min via i.p. **f**–**g** qPCR analysis of the mRNA level of proinflammatory cytokines in inguinal fat pads of HFD mice (*n* = 8/group) and *ob/ob* mice (*n* = 6/group). Statistical analysis was performed by collating the results from multiple samples. *P < 0.05, **P < 0.01, ***P < 0.001

To explore whether downregulation of B4GalT5 affect obesity-associated metabolic status, we knocked down B4GalT5 in HFD-fed mice and *ob/ob* mice by injecting adenovirus carrying B4GalT5 shRNA into both sides of inguinal fat pads. Interestingly, blood glucose levels were decreased by B4GalT5 knockdown under fasting and fed conditions (Fig. 2a, b). In addition, intraperitoneal glucose tolerance test (GTT) and insulin tolerance test (ITT) showed B4GalT5 knockdown mice were protected from obesity-induced glucose intolerance and insulin resistance (Fig. 2c, d). The insulin-signaling pathway in subcutaneous adipose tissue was intensified by B4GalT5 knockdown (Fig. 2e). Furthermore, cholesterol and low-density lipoprotein cholesterol (LDL-c) also improved in the B4GalT5 knockdown HFD-fed mice (Supplementary Fig. A). Because obesity is associated with low-grade chronic inflammation in adipose tissue and inflammation plays a pivotal role in the pathogenesis of insulin resistance and T2D, we detected the expression of some proinflammatory cytokines. IFN-γ, TNF-α, and IL-6 were all downregulated in B4GalT5 knockdown HFD mice and *ob/ob* mice (Fig. 2f, g). These results indicated B4GalT5 knockdown in subcutaneous adipose tissue protected mice from obesity-induced insulin resistance and adipose inflammation.

### B4GalT5 knockdown in subcutaneous adipose tissue increased adipogenesis

**Fig. 3:**
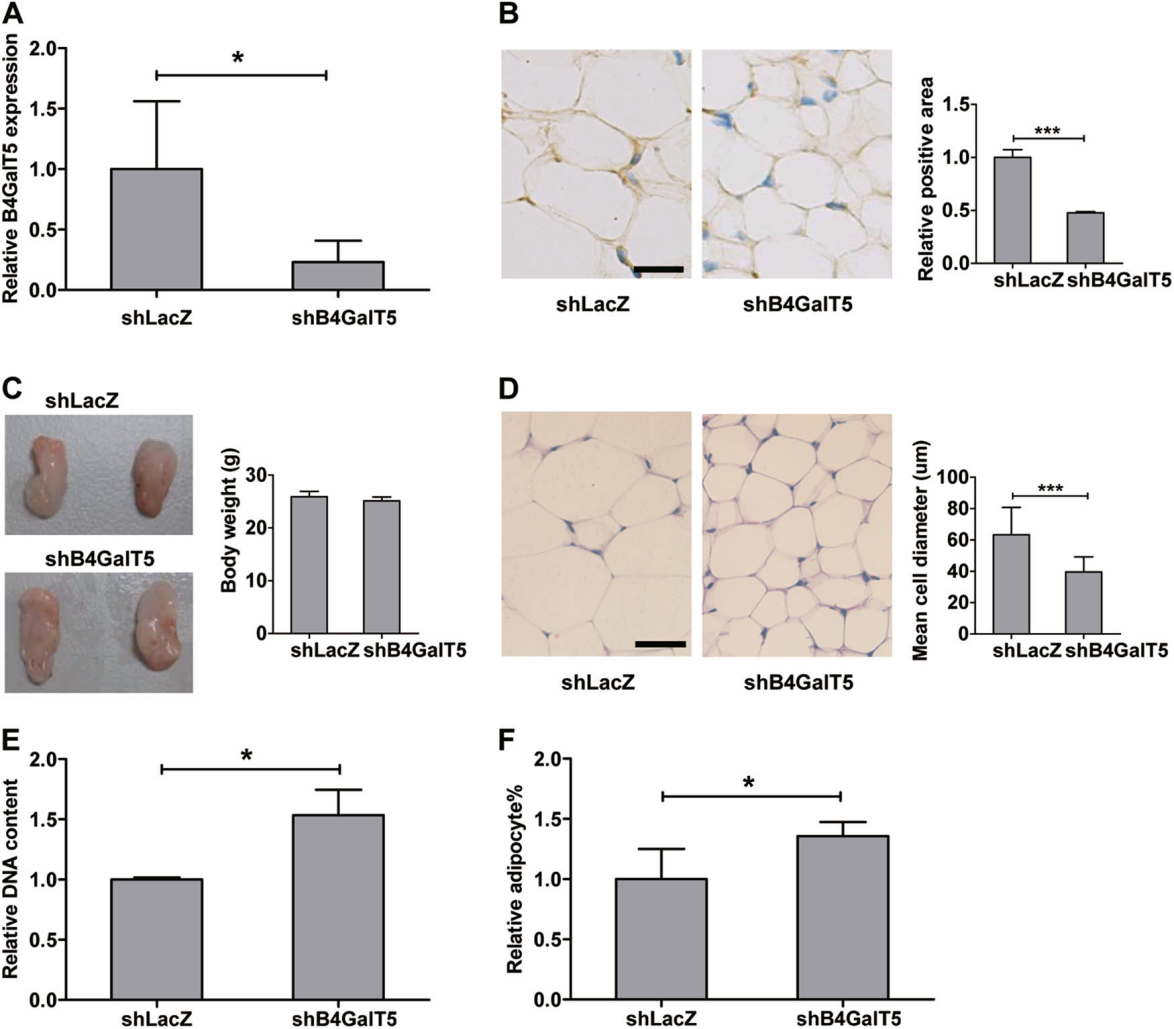
Downregulation of B4GalT5 increased adipogenesis in subcutaneous adipose tissue. Six-week-old C57BL/6 J mice were injected with adenovirus expressing B4GalT5 or control LacZ shRNA twice a week s.c. adjacent to both sides of the inguinal fat pads for 6 weeks. Both groups of mice were fed with HFD. **a** Knockdown of B4GalT5 expression in inguinal fat pads was confirmed by qPCR (*n* = 8/group). **b** IHC staining for B4GalT5 was conducted in the inguinal fat pads of mice treated with B4GalT5 or LacZ shRNA adenovirus (Scale bar: 20 μm). B4GalT5 IHC results were quantified by Image pro plus 6.0 image analysis software. **c** Comparison of inguinal fat pads in mice treated with B4GalT5 or LacZ shRNA adenovirus. Fat pads were weighed and statistically analyzed. **d** Representative H&E staining of inguinal fat pads in mice treated with B4GalT5 or LacZ shRNA adenovirus. Scale bar: 20 μm. Cell diameter was measured in the H&E-stained sections of three individual samples in each group. **e** Relative DNA amount of the whole inguinal fat pads from mice treated with B4GalT5 or LacZ shRNA (*n* = 4). **f** The percentage of mature adipocytes relative to total cell number in inguinal fat pads was statistically analyzed by flow cytometry between the two groups (*n* = 8). Statistical analysis was performed by collating the results from multiple samples. *P < 0.05, **P < 0.01, ***P < 0.001

Downregulation of B4GalT5 in inguinal fat pads of HFD-fed mice did not affect the body weight (Supplementary Fig. B) or subcutaneous adipose tissue weight (Fig. 3c). Knockdown of B4GalT5 expression in inguinal fat pads was confirmed by qPCR and immunohistochemistry assay (Fig. 3a, b). However, the adipocyte size was much smaller in the inguinal fat pads of the B4GalT5 knockdown mice than that in the control mice (Fig. 3d). The adipocyte number (based on the DNA content and the percent of mature adipocytes relative to total cell number in the inguinal fat pads) were higher in B4GalT5-knockdown mice (Fig. 3e, f). This indicated that adipogenic commitment and differentiation increased in B4GalT5 knockdown mice compared with that in the control.

### Downregulation of B4GalT5 promoted adipogenic commitment by activating BMP signaling pathway

**Fig. 4:**
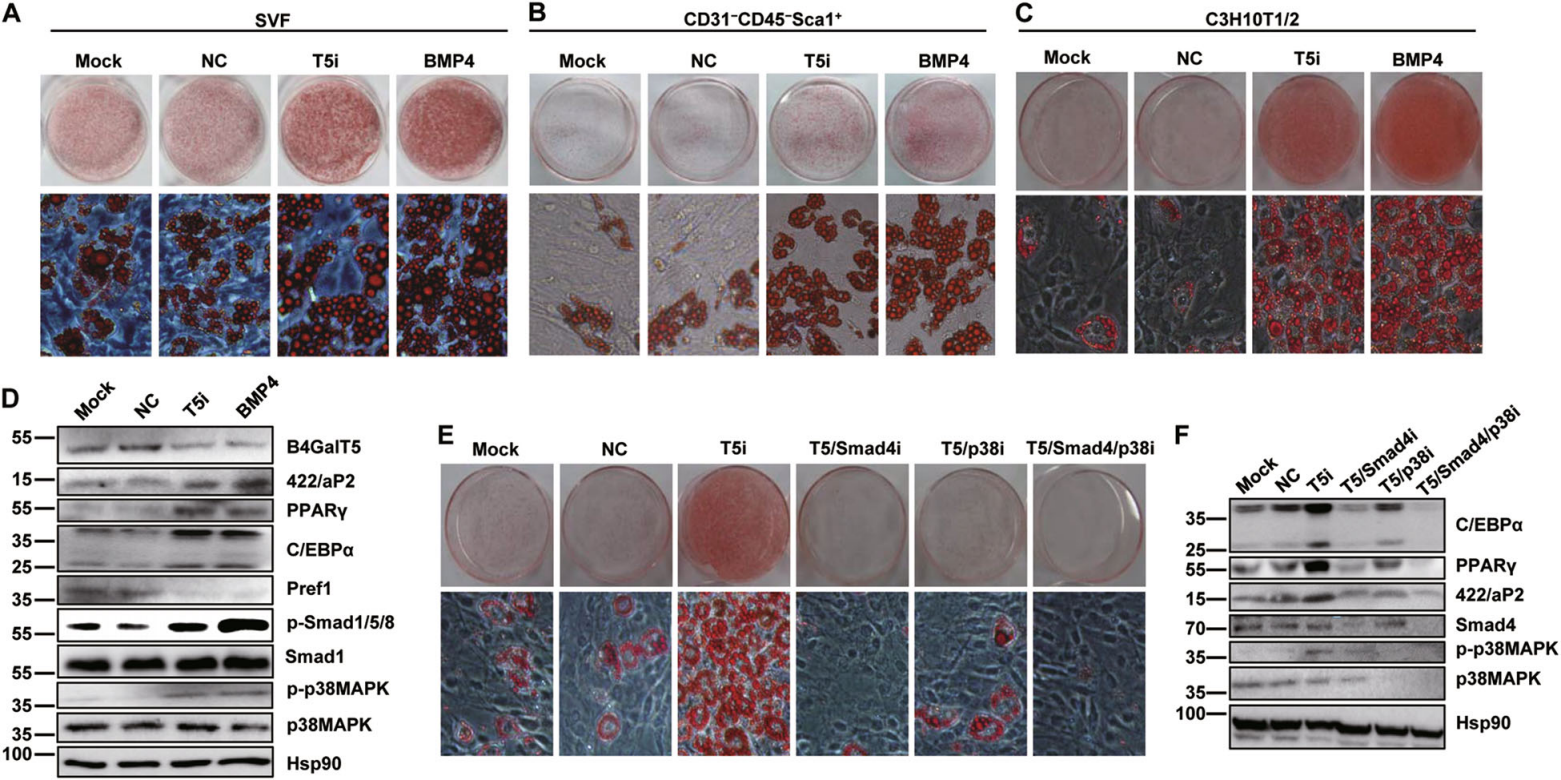
B4GalT5 knockdown promotes commitment of C3H10T1/2 cells to adipocyte lineage probably through BMP signaling. **a**–**c** The effect of B4GalT5 knockdown on adipogenic commitment and subsequent differentiation was assessed by oil red O staining on day 6 after MDI induction. Mock, blank control; NC, negative control, treated with negative scramble siRNA; T5i, experimental group, treated with B4GalT5 siRNA; BMP4, positive control. **a** Oil red O staining of SVF cells. **b** Oil red O staining of adipocyte-progenitor cells. **c** Oil red O staining of C3H10T1/2 cells. **d** Western blotting of PPARγ, C/EBPα, and 422/aP2 on day 6 after induction was performed to analyze adipogenesis, protein level of B4GalT5 was also shown. Western blot analysis of the BMP signaling pathways on day 0 using the antibodies indicated above, protein levels of B4GalT5 and Pref1 were also shown, with Hsp90 as a loading control. **e** On day 6 post-induction, cells were stained with oil red O to assess adipogenesis. **f** Knockdown effect of smad4 and p38 MAPK were confirmed by western blotting on day 0, and western blotting of PPARγ, C/EBPα, and 422/aP2 on day 6 post-induction. Each data is representative of at least three independent experiments with identical results. Each data is representative of at least three independent experiments with identical results. *P < 0.05, **P < 0.01, ***P < 0.001

To further explore the effect of B4GalT5 downregulation on adipocyte development, we examined the function of B4GalT5 on adipogenesis with cell models. Since B4GalT5 is mostly located in SVF rather than mature adipocytes (Fig. 1e, f), we knocked down B4GalT5 by RNAi on SVF and observed that adipogenesis of SVF was promoted (Fig. 4a). Since SVF contains various stages of preadipocyte, we speculated that B4GalT5 might influence the adipogenic commitment and differentiation. First, we intervened B4GalT5 expression in 3T3-L1 preadipocytes and found that B4GalT5 had little effect on the terminal differentiation (data not shown). Then we isolated adipocyte-progenitor cells (CD31^−^CD45^−^Sca1^+^ cells) from SVF using MACS magnetic beads. As expected, downregulation B4GalT5 promoted adipocyte commitment and differentiation of adipocyte-progenitor cells ex vivo, as illustrated by Oil Red O staining (Fig. 4b). Furthermore, we confirmed it on C3H10T1/2 cells, which were one of the faithful models in vitro for long-term genetic studies of the adipocyte developmental program^7^. We found that downregulation of B4GalT5 induced commitment of C3H10T1/2 cells to the adipocyte lineage even in the absence of bone morphogenic protein 4 (BMP4), which was required for adipogenic commitment^30^. The increased expression of mature adipocyte markers of peroxisome proliferator-activated receptor-γ (PPARγ), CCAAT/enhancer-binding protein-α (C/EBPα) and 422/aP2 and the reduced expression of preadipocyte factor-1(Pref1) also indicated the enhancement of adipogenic commitment when B4GalT5 was downregulated (Fig. 4c, d).

To explore the pathways involved in B4GalT5-mediated adipogenesis, we detected key molecules in the signaling pathways involved in adipogenic commitment, such as BMP, Wnt, Hedgehog pathway^31^. B4GalT5 knockdown led to activation of Smad-dependent (phosphorylation of Smad1/5/8) and Smad-independent (phosphorylation of p38 MAPK) BMP signaling as BMP4 treated positive control (Fig. 4d). When knocked down Smad4 (coregulator in the BMP/Smad signaling pathway) or/and p38 MAPK simultaneously with B4GalT5 by RNAi in C3H10T1/2 cells, we found that both Smad4 and p38 MAPK were required for B4GalT5-mediated adipogenic commitment (Fig. 4e, f). Wnt signaling pathway also downregulated (Supplementary Fig. C–F), probably resulted from the crosstalk between Wnt and BMP pathways^32^. These results suggested that downregulation of B4GalT5 promoted commitment of C3H10T1/2 cells to the adipocyte lineage, probably through BMP signaling pathways.

### B4GalT5 regulated the stability and intracellular distribution of BMPRIA

**Fig. 5:**
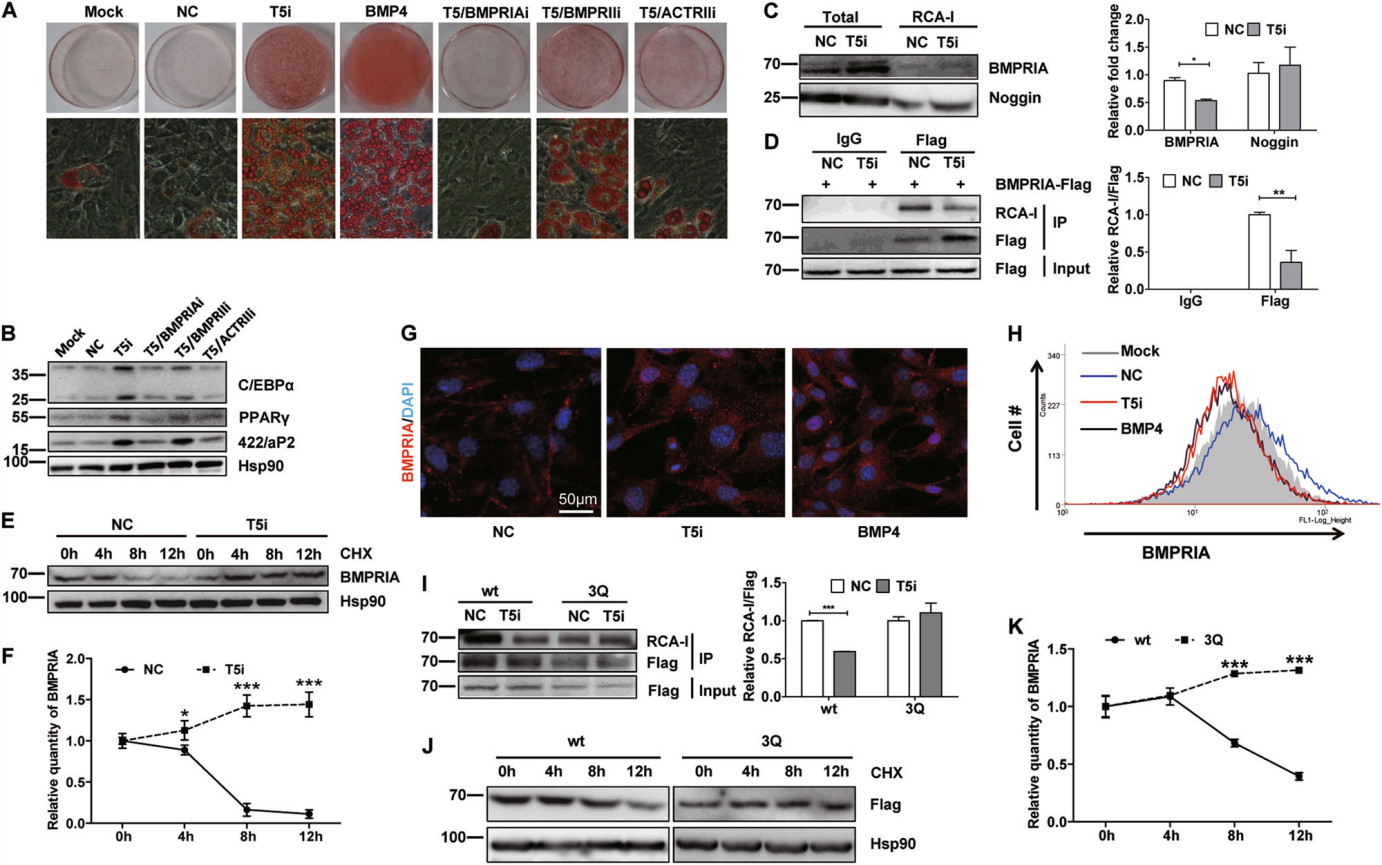
B4GalT5 knockdown reduces BMPRIA glycosylation, increases its stability and enhances its intracellular distribution. **a** On day 6 post-induction, cells were stained with oil red O to assess adipogenesis. **b** Western blotting of PPARγ, C/EBPα, and 422/aP2 on day 6 post-induction. **c** RCA-I pulldown assay to analyze BMPRIA and noggin glycosylation level in C3H10T1/2 cells with different treatment. Results were quantified; the fold change describes the change in the proportion of bound divided by total for each protein. **d** C3H10T1/2 cells treated with NC or B4GalT5 siRNA were then transfected with wild-type Flag-BMPRIA plasmids. Forty-eight hours later, coimmunoprecipitation assays were performed with anti-Flag antibody, followed by lectin blotting assay to detect the RCA-I-binding level. Results were quantified; the fold change describes the change in the proportion of bound RCA-I level divided by the pull-downed Flag. **e** C3H10T1/2 cells treated with B4GalT5 siRNA or negative control were incubated with cycloheximide (40 μM) for the indicated times on day 0. After treatment, cell lysates were checked by western blotting with anti-BMPRIA antibody. **f** The BMPRIA protein level after cycloheximide treatment in panel** e** was quantified, normalization to 0 h, and plotted as shown. **g** C3H10T1/2 cells with different treatment were performed immunofluorescence staining to demonstrate BMPRIA distribution in the cellular level (Scale bar: 50 μm). **h** BMPRIA cell surface expression was displayed by flow cytometric analysis. **i** C3H10T1/2 cells treated with NC or B4GalT5 siRNA were then transfected with wild-type Flag-BMPRIA or 3Q mutant Flag-BMPRIA plasmids. Forty-eight hours later, coimmunoprecipitation assays were performed with anti-Flag antibodies, followed by lectin blotting assay to detect the RCA-I-binding level. Results were quantified; the fold change describes the change in the proportion of bound RCA-I level divided by the pull-downed Flag. **j** C3H10T1/2 cells were transfected with wild-type Flag-BMPRIA or 3Q mutant Flag-BMPRIA plasmids, 48 h later, cells were incubated with CHX for the indicated times. Cell lysates were monitored by western blotting with anti-Flag antibody. **k** The Flag levels in Fig. 5J were quantified, normalization to 0 h and plotted as shown. Each data is representative of at least three independent experiments with identical results. *P < 0.05, **P < 0.01, ***P < 0.001

BMP signaling pathway is activated through two distinct type 1 and 2 receptors. C3H10T1/2 cells express the type 1 receptor BMPRIA and the type 2 receptors BMPRII and ACTRII^33^. We lowered their expression simultaneously with B4GalT5 by RNAi. The adipogenesis-promoting effect of B4GalT5 downregulation was completely (BMPRIA RNAi) or partially (BMPRII or ACTRII RNAi) eliminated. The adipogenic commitment and terminal differentiation of C3H10T1/2 was impaired, as indicated by the accumulation of cytoplasmic triglycerides and expression of PPARγ, C/EBPα, and 422/aP2 (Fig. 5a, b). These results illustrated that B4GalT5 downregulation induced adipocyte commitment through both type 1A and 2 BMP receptors.

Lectin ricinus communis agglutinin I (RCA-I) specifically binds to and interacts with oligosaccharides terminating with the Gal β1 → 4GlcNAc group. To identify potential B4GalT5 target genes during adipocyte commitment, we performed RCA-I lectin blotting assays in C3H10T1/2 cells. The 35–70 kDa and 250 kDa around glycoproteins decreased their binding with RCA-I significantly in the B4GalT5 knockdown group relative to control cells (Supplementary Fig. G). As BMP signal activation was required for B4GalT5-mediated adipocyte commitment in C3H10T1/2 cells, we examined whether B4GalT5 modified the glycan structures of the key components of the BMP pathway, including the BMP ligands, receptors, and BMP antagonists. No significant differences were observed between the B4GalT5 knockdown and control cells in the binding of RCA-I with most of these factors (such as noggin). However, the relative level of BMPRIA pulled-down by RCA-I was much less in B4GalT5 knockdown cells than in control (Fig. 5c). Meanwhile, we pulled down BMPRIA and detected RCA-I binding level in B4GalT5 knockdown and control cells. We found that the RCA-I-binding level of Flag-BMPRIA was decreased significantly with B4GalT5 knockdown (Fig. 5d). This indicated B4GalT5 might modify the glycan structures of BMPRIA.

We noticed that the total BMPRIA protein level increased in B4GalT5 knockdown cells (Fig. 5c). Previous studies indicate galactosyltransferase activity and glycosylation level may influence cell surface half-life of the membrane receptor, coincident with the endocytosis and redistribution of the receptor^16^. To investigate the stability of BMPRIA, protein synthesis was blocked with cycloheximide. BMPRIA stability increased markedly with B4GalT5 knockdown (Fig. 5e, f). We then performed immunofluorescence (Fig. 5g) and flow cytometry (Fig. 5h) to examine the distribution of BMPRIA in C3H10T1/2 cells treated with B4GalT5 siRNA. The cell surface BMPRIA level was decreased and the intracellular distribution of BMPRIA increased subsequently with B4GalT5 downregulation. Since internalization of BMP receptors is highly related to the activation of BMP signaling, these data suggested B4GalT5 knockdown decreased BMPRIA glycosylation level, increased BMPRIA stability and caused redistribution of BMPRIA.

B4GalT5 is generally responsible for the synthesis of complex-type *N*-linked oligosaccharides in many glycoproteins^1,9^, we screened the amino-acid sequences of BMPRIA and found three potential *N*-linked glycosylation sites (N73, N314, and N373). To confirm BMPRIA is the target of B4GalT5, we mutated all these sites to glutamine (3Q) synchronously and over-expressed wild-type or the mutant BMPRIA in C3H10T1/2 cells. As expected, the glycosylation level (demonstrated by the RCA-I binding level after pulled down by Flag) of the 3Q mutant remained unchanged with B4GalT5 knockdown (Fig. 5I). Also, compared with the wild-type BMPRIA, the 3Q mutant increased its stability significantly (Fig. 5j, k). These data indicated BMPRIA was modified by B4GalT5 and B4GalT5 knockdown increased its stability and thus activating BMP signaling.

### Downregulation of B4GalT5 depress M1 infiltration in subcutaneous adipose tissue

**Fig. 6:**
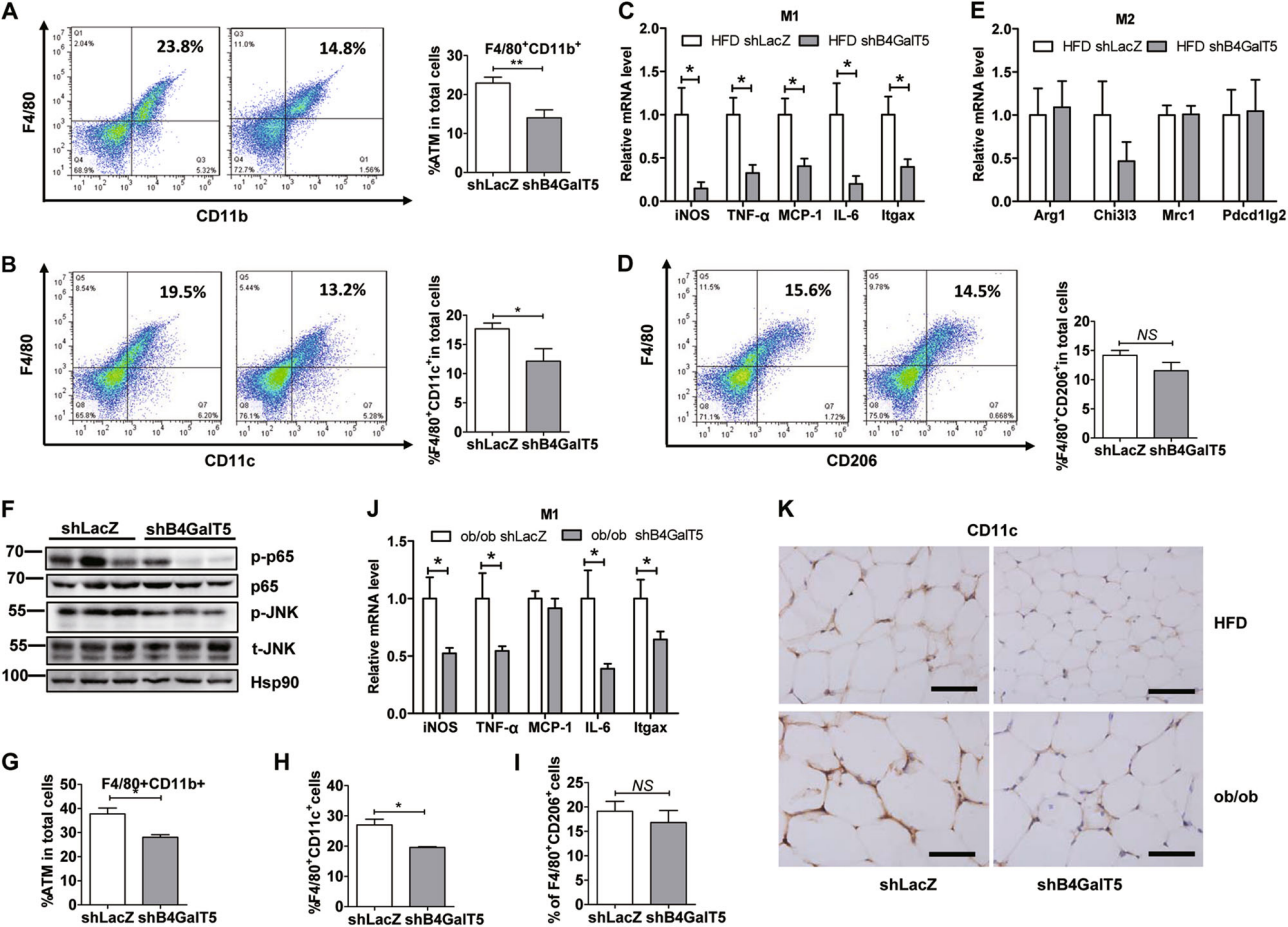
Downregulation of B4GalT5 reduce HFD-induced M1 macrophage infiltration in subcutaneous adipose tissue. Six-week-old C57BL/6 J mice and *ob/ob* mice were injected with adenovirus expressing B4GalT5 or control LacZ shRNA twice a week s.c. adjacent to both sides of the inguinal fat pads for 6 weeks. HFD feeding started at 6 weeks of age on WT mice. **a**–**f** HFD mice; **g**–**j*** ob/ob* mice. **a** The adipose macrophages (CD11b^+^F4/80^+^) were examined by flow cytometry analysis from SVF. **b**, **d** FACS analysis determined iWAT F4/80^+^CD11c^+^M1and F4/80^+^CD206^+^ M2 macrophages (*n* = 6/group). **c**, **e** qPCR determined M1 markers, iNOS, TNF-α, MCP-1, IL-6, Itgax, and M2 markers, Arg1, Chi3l3, Mrc1, Pdec1lg2 in iWAT from different mouse group as indicated (*n* = 6/group). **f** Western blotting analysis of the p-JNK, total(t)-JNK, p-p65, and p65 in the iWAT. **g**–**i** FACS analysis determined iWAT total macrophages (CD11b^+^F4/80^+^), F4/80^+^CD11c^+^M1and F4/80^+^CD206^+^ M2 macrophages. **j** mRNA level of M1 markers. **k** CD11c IHC of iWAT sections (five images per mouse), scale bars 20 µm. Each data is representative of at least three independent experiments with identical results. *P < 0.05, **P < 0.01, ***P < 0.001

B4GalT5 knockdown in subcutaneous adipose tissue protected mice from obesity-induced insulin resistance and adipose inflammation (Fig. 2). Macrophage is an important initiator of the adipose tissue inflammatory reaction during obesity^14^, moreover, B4GalT5 expression is higher in macrophage than adipocytes in mice (BioGPS), we next explore whether B4GalT5 might be coupled to metabolic inflammation by influencing macrophage. Quantification of FACS data revealed a significant reduce of macrophage (CD11b^+^F4/80^+^) infiltration in the inguinal fat pads of the B4GalT5 knockdown mice after HFD (Fig. 6a). Of note, the proportion of M1 (F4/80^+^CD11c^+^) in SVF from B4GalT5 knockdown mice was significantly decreased (Fig. 6b); consistent with results of immunohistochemistry (Fig. 6k). Because M1 macrophages contribute to insulin resistance by producing inflammatory cytokines that impaired insulin-signaling pathway^34^, we next evaluated the expression of M1 macrophage marker genes, including iNOS, TNF-a, MCP-1, IL-6, and Itgax in iWAT. Notably, proinflammation factors were drastically reduced in B4GalT5 downregulation mice (Fig. 6c). This was accompanied by attenuated activation of nuclear factor-κB p65 and JNK phosphorylation, which are reportedly involved in regulating obesity-induced inflammation and insulin resistance, in B4GalT5 downregulation mice compared with controls (Fig. 6f). Although the M2 (F4/80^+^CD206^+^) macrophage content did not change (Fig. 6d, e), we affirmed that downregulation of B4GalT5 attenuated M1 macrophages infiltration in HFD-fed mice. Consistently, when we knocked down B4GalT5 in *ob/ob* mice, we got the consistent results (Fig. 6g–k). Altogether, in both HFD mice and *ob/ob* mice, downregulation of B4GalT5 depressed the infiltration of M1 macrophages and reduced adipose inflammation.

### Downregulation of B4GalT5 restrain proinflammatory factors in macrophages

**Fig. 7:**
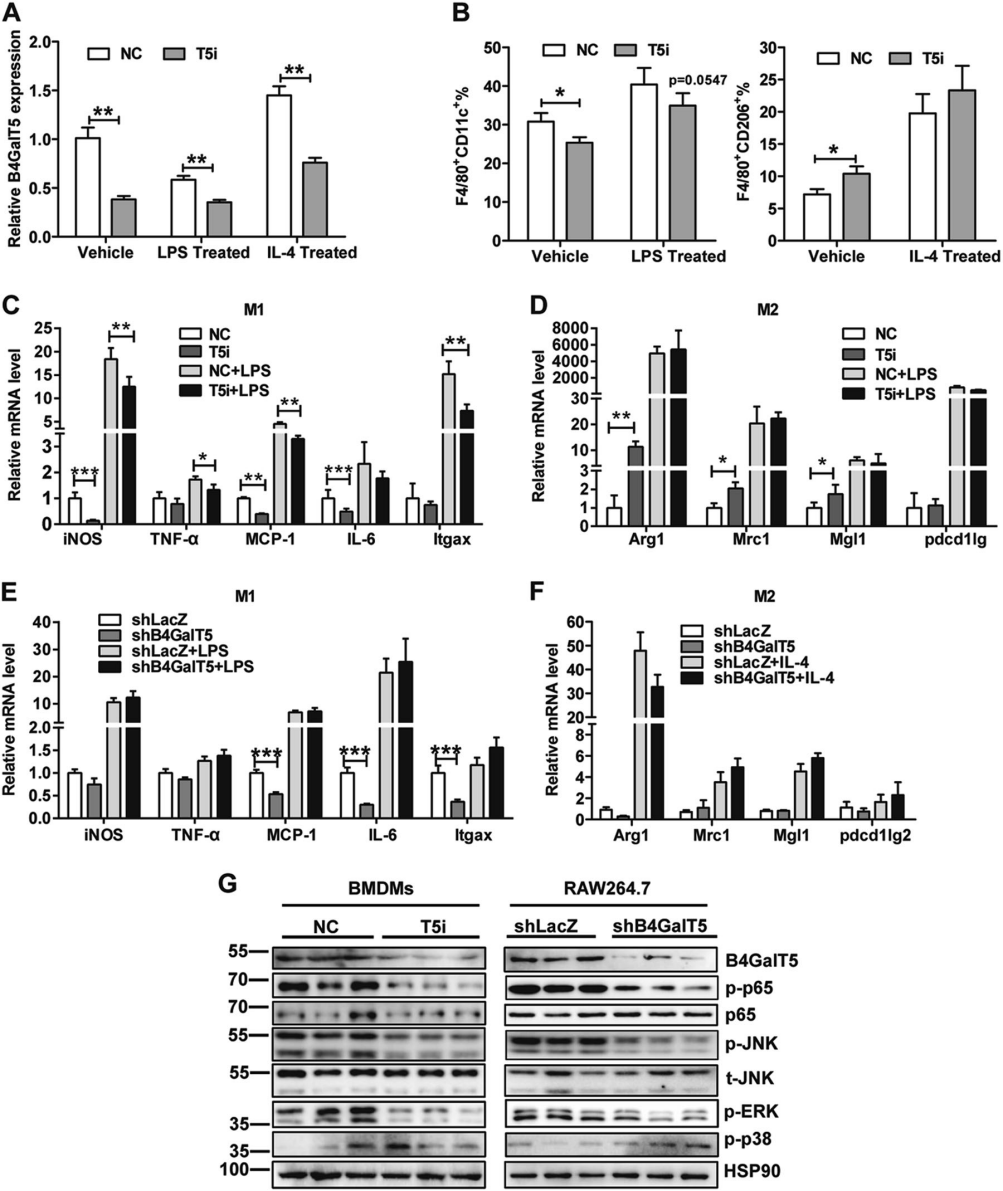
Effects of downregulation of B4GalT5 on macrophage. BMDMs from 6-week-old C57BL/6 J mice were treated with 100 ng/ml LPS or 10ng/ml IL-4 or vehicle control for 24 h. B4GalT5 was silenced by siB4GalT5 in BMDMs, downregulation of B4GalT5 by shRNA in RAW264.7 macrophages. **a** qPCR analysis of the mRNA level of B4GalT5 on different groups. **b** Flow cytometry analysis of the expression of F4/80, CD11c, and CD206. Percentages of F4/80^+^CD11c^+^ cells and F4/80^+^CD206^+^ cells are shown. **c** Relative mRNA abundance of M1 macrophage marker genes. **d** Relative mRNA abundance of M2 macrophage marker genes. **e** Relative mRNA abundance of M1 macrophage marker genes. **f** Relative mRNA abundance of M2 macrophage marker genes. **g** Western blotting was conducted to measure the level of the indicated protein. Each data is representative of at least three independent experiments with identical results. *P < 0.05, **P < 0.01, ***P < 0.001

In obesity, infiltrated macrophages in adipose tissue were mainly bone marrow-derived macrophages (BMDMs)^35^. To confirm the direct effect of B4GalT5 on macrophage, we isolated the bone marrows from lean mice and cultured to undifferentiated mature macrophages M0 after 7 days of induction with M-CSF. When we downregulated B4GalT5 by RNAi (Fig. 7a), FACS analysis showed the ratio of M1 (F4/80^+^CD11c^+^) macrophages decreased and M2(F4/80^+^CD206^+^) macrophages increased (Fig. 7b). The mRNA of M1 marker genes were greatly reduced, such as iNOS, TNF-a, MCP-1, and IL-6 (Fig. 7c). Meanwhile, the expression of M2 markers were increased, including Arginase 1, Chi3l3, and Mrc1 (Fig. 7d). Furthermore, we downregulated B4GalT5 by shRNA in RAW264.7 macrophages, we found the decrease of M1 marker genes (Fig. 7e) and no change on M2 marker genes (Fig. 7f). Of note, when B4GalT5 was knocked down, the activation of JNK and NFκB/p65 were inhibited both in BMDMs and RAW264.7 macrophages (Fig. 7g). Collectively these results indicate that downregulation of B4GalT5 suppress M1 macrophage-mediated adipose tissue inflammation by repressing JNK and NFκB/p65 activation, which are major pathways involved in inflammatory factors expression in macrophages^36,37^.

## Discussion

Aberrant expression of carbohydrate structures has been observed in many metabolic disorders^38,39^. Conditional regulation of *N*-glycan processing, for example, interfering with expression of a particular glycosyltransferase, may help maintain normal metabolic activities. We found for the first time that downregulation of B4GalT5 improved obesity-induced insulin sensitivity through regulating adipogenesis and M1 macrophage infiltration. Interestingly, B4GalT5 is included in a locus that has been reproducibly identified to be linked with obesity in genome-wide linkage studies^40^.

Although the body weight and size of inguinal fat pads were similar between B4GalT5 knockdown and control mice, metabolic status (insulin sensitivity, adipose tissue inflammation, and blood biochemistry markers) was improved in the B4GalT5 knockdown mice. We observed that adipocyte size was smaller and adipocyte number increased after downregulation of B4GalT5 in subcutaneous adipose tissue. Although the increase in adipocyte number is well tolerated, the growth in size is sensed as a harmful process, because adipocyte hypertrophy is closely related to adipocyte dysfunction and inflammation^41^. In this case, the improvement in metabolic status in B4GalT5 knockdown mice might be resulted from resisting hypertrophy through enhancing adipogenesis. The phenotype of reducing adipocyte size and improving metabolic status in B4GalT5 knockdown mice were similar to the fatty acid-binding protein 4 (Fabp4) promoter-driven adipose tissue-specific BMP4 transgenic mice^42^. But the mechanism was totally different, in BMP4 transgenic mice the browning of subcutaneous adipose tissue was enhanced, but the beige-specific genes (such as CD137, TMEM26, and Tbx1) did not change significantly in B4GalT5 knockdown mice (unpublished data).

We found downregulation of B4GalT5 promoted adipogenesis by activating BMP signaling. Our results are consistent with a previous study showing that B4GalT5 is required for proper patterning of the dorsoventral axis during zebrafish embryogenesis through the regulation of BMP signaling^31^. Studies demonstrated that receptor glycosylation modulates the endocytosis and cellular distribution of cell surface glycoproteins by binding to lectins and sterically modulating molecular interactions, thereby controlling threshold for cell signaling^15,43^. B4GalT5 knockdown reduced BMPRIA glycosylation, thereby promoting BMP signaling transduction by increasing stability and intracellular distribution of the receptor. Mutant of the BMPRIA *N*-glycosylation sites also enhanced the stability of BMPRIA. This suggested BMPRIA glycosylation was closely correlated with its stability. The mechanism responsible for the BMPRIA glycosylation and its stability and cellular redistribution is now unclear. One hypothesis is BMPRIA glycosylation alters the *N*-glycan structure of it, interferes with the binding of BMPRIA with some lectins and proteins, and thus affects the distribution of BMPRIA at the cellular level. BMPRIA glycosylation might also influence the phosphorylation level of BMPRIA, which activates BMPRIA and downstream BMP signaling. Further investigation is required to fully understand the mechanisms through which B4GalT5 modulates glycosylation and cellular distribution of BMPRIA.

The apparent number of early progenitor cells in the subcutaneous adipose tissue that can undergo differentiation, which is reduced in obesity and the differentiation capacity negatively correlated with BMI^44^. While, the expression of B4GalT5 was positively correlated to BMI, so B4GalT5 might be one of the negative regulators of adipogenesis in obesity and our results also supported the hypothesis. Furthermore, the high expression of B4GalT5 in obesity may be related to the infiltration of macrophages. And inflammatory cytokines induced a proinflammatory and macrophage-like phenotype of the preadipocytes and restrain adipogenesis^45^. Meanwhile, it is reported that TNF-α elevated expression of B4GalT5 in 3T3-L1 cells^29^. We hypothesized that inflammatory cytokines might depress adipogenesis by upregulating B4GalT5 expression in preadipocytes. Further studies are required to confirm the relationship between inflammatory cytokines and expression of B4GalT5 in preadipocytes.

NFκB and JNK pathways are one of the major pathways that can be involved in inflammation-induced insulin resistance^46^. Recently, studies have suggested that JNK pathway is involved in impaired insulin signaling through induced serine phosphorylation of IRS-1 at Ser307^47^. Our study also shows downregulation of B4GalT5 repressed the activation of these inflammatory signaling pathways both in the subcutaneous adipose tissue and BMDMs and RAW264.7 macrophages. Further studies are needed on how these signaling pathways were regulated through glycosylating on targets of B4GalT5.

Collectively, these studies provided new insight into the effects of B4GalT5 on the commitment of adipocytes and activation of M1 macrophages in obesity. B4GalT5 may be a regulator of inflammatory cytokines and M1 infiltration, which contribute to insulin resistance in adipose tissue. Thus, B4GalT5 might be a new potential target for reducing obesity and insulin resistance.

## Materials and methods

### Human adipose tissue samples

Human B4GalT5 studies were conducted using subcutaneous adipose tissues and measurements of BMI (16.7–42.2 kg/m^2^), and T2D status from patients who underwent surgery irrelevant to metabolic disease in Shanghai Jiao tong University Affliated Sixth and Ninth People’s Hospital. BMI of obese subjects was over 30 kg/m^2^. This study was approved by the ethics committees of Fudan University Shanghai Medical College and was in accordance with the principle of the Helsinki Declaration II. Written informed consent was obtained from each participant.

### Mice stud**i**es

Four- to six-week-old male C57BL/6 J mice were purchased from the Experimental Animal Center of Chinese Academy of Sciences (Shanghai, P.R. China). Mice were fed with normal chow or an HFD (60% k cal in fat, beginning at age 6 week). Five-week-old *ob/ob* mice were purchased from the Model Animal Research Center of Nanjing University. These mice were maintained on normal chow diet (ND). All studies were approved by the Animal Care and Use Committee of the Fudan University Shanghai Medical College and followed the National Institute of Health guidelines on the care and use of animals.

### Cell culture and induction of differentiation

C3H10T1/2 cells were cultured in Dulbecco’s modified Eagle’s medium (DMEM) containing 10% calf serum with or without 20 ng/ml purified recombinant BMP4 (R&D Systems). To induce differentiation, 2-day post-confluent cells (designated day 0) were provided with DMEM containing 10% fetal bovine serum (FBS), 1 μg/ml insulin (I), 1 μM dexamethasone (D), and 0.5 mM 3-isobutyl-1-methyl-xanthine (M) for 2 days. Cells were then fed with DMEM supplemented with 10% FBS and 1 μg/ml insulin for another 2 days, after which time they were fed every other day with DMEM containing 10% FBS.

### Isolation of SVF and adipocytes from adipose tissue and SVF culture

Adipose tissue was harvested and isolated by enzymatic digestion (collagenase VIII; Sigma). The digested tissue was filtered through a 100-μm mesh filter to remove debris and was centrifuged. The adipocytes floated above the supernatant. The cellular pellet involving the SVF was resuspended with an ammonium chloride lysis buffer to remove red blood cells. Both SVF and adipocytes were washed with 0.5% calf serum in phosphate-buffered saline (PBS). The whole SVF was cultured as described previously^48^.

### Adipocyte-progenitor cells isolation and adipocyte differentiation

For adipocyte-progenitor-cell isolation, SV cells from subcutaneous adipose tissue from lean C57BL/6 mice were washed with PBS containing 0.5% BSA (Sigma-Aldrich), incubated with Fc block (Bioscience) and stained with anti-CD31 (FITC, Biolegend) for 20 min at 4 °C. CD31-positive cells were removed by using MACS magnetic beads (anti-FITC, Miltenyi). Afterward, the SV cells underwent CD45 positive selection to remove hematopoietic cells using MACS magnetic beads (anti-CD45, Miltenyi). Adipocyte progenitors were sorted as CD31^−^CD45^−^Sca1^+^ cells. The cells were resuspended in DMEM/F12 medium supplemented with 10% FBS. Then they were seeded into culture dishes until they reached 70–80% confluence.

For the differentiation of adipocyte progenitors into adipocytes, cells were seeded into 6-well plates at a density of 5 × 10^4^ cells per well in 2 ml complete DMEM/F12. When the cells reached confluence (day 0), cell medium was replaced with medium containing insulin (0.5 µg/ml, Sigma-Aldrich), 3-isobutyl-1-methylxantine (0.5 mM, Sigma-Aldrich), dexamethasone (1 µM), pioglitazone (1 µM, Sigma-Aldrich). After 48 h, medium was exchanged with complete DMEM/F12 containing insulin (0.5 µg/ml) and pioglitazone (1 µM). Differentiated adipocytes (days 8–10) were used for assays.

### Isolation of BMDMs and culture

To isolate BMDMs, four- to six-week-old male C57BL/6 J mice were broken cervical vertebra, and their femur and tibias were collected. Bone marrow were cultured and differentiated for 7 days, in DMEM medium supplemented with 10% fetal bovine serum (FBS), 1% penicillin/streptomycin (P/S), and M-SCF(10ng/ml). Macrophages were treated with 10 ng/ml LPS or IL-4 for 24 h for gene expression analysis and flow cytometer analysis.

### RNA isolation and real-time quantitative PCR

Total RNA was isolated using TRIzol reagent (Invitrogen, Carlsbad, CA). Quantitative PCR (qPCR) involved Power SYBR green PCR master mix (Applied Biosystems, Carlsbad, CA) and a Prism 7500 instrument (Applied Biosystems), with GAPDH mRNA as an endogenous control. Analysis was done in triplicate and repeated at least three times. Results were presented as means and standard deviations (SD) from three independent experiments. Details of primers are available on request.

### Glucose and insulin tolerance tests

For the GTT, mice were injected i.p. with d-glucose (2 mg/g body weight) after an overnight fast, and tail blood glucose levels were monitored at the indicated time. For the ITT, mice fed ad libitum were injected i.p. with human insulin (Eli Lilly) (0.75 mU/g body weight) at around 2 p.m., and tail blood glucose levels were monitored at the indicated time.

### RNA interference

C3H10T1/2 cells or mature macrophage (derived from bone marrow) were transfected with siRNA using Lipofectamine RNAiMAX (Invitrogen) according to the manufacturer’s instructions. The sequences used for successful B4GalT5 knockdown were kindly provided by Jian-Hai Jiang, UGUCACGUACGACGCCUUG. Stealth RNAiTM siRNA duplexes specific for Smad4, p38 MAPK were synthesized by Invitrogen. The sequences are as follows:

Smad4, CAUACACACCUAAUUUGCCUCACCA;

p38 MAPK, CCUUUGAAAGCAGGGACCUUCUCAU

### H&E staining and cell size quantization

Standard H&E staining was performed on 5-μm paraffin sections of inguinal adipose tissue. The expression of B4GalT5 was detected by immunohistochemistry (IHC) using its antibody (sc-22291-R, 1:500). Adipose tissue macrophage infiltration was evaluated by IHC using a CD11c antibody (Abcam). Cell diameter was measured in the H&E-stained sections of three individual samples in each group using Image J.

### Measurements of blood parameters

Mice with different treatment were fasted overnight, and blood samples were collected by retroorbital bleeding methods. Sera were prepared and used for measurements. Triglycerides, LDL, high-density lipoproteins (HDL), and cholesterol levels were determined using the CobasC311 automatic biochemical analysis device (Roche).

### Oil red O staining for lipid

In vitro differentiated cells were fixed for 20 min in buffered formalin and stained with oil red O for 60 min. The stained fat droplets in the cells were visualized by light microscopy and photographed.

### Western blotting

Cells were scraped into lysis buffer containing 2% sodium dodecyl sulfate (SDS) and 50 mM Tris–HCl (pH 6.8). Lysates were quantitated and equal amounts of protein were subjected to sodium dodecyl sulfate polyacrylamide gel electrophoresis (SDS–PAGE). Proteins were then transferred onto Polyvinylidene fluoride (PVDF) membrane and immunoblotted with specific antibodies. Antibodies used were PPARγ, phosphor-p38 MAPK, p38 MAPK kinase, phosphor-Smad1/5/8, Smad1, Smad4, Pref1, phosphor-Akt, Akt, phosphor-ERK, ERK, phosphor-JNK, JNK, phosphor-p65, p65 (Cell Signaling Technology), BMPRIA (Abcam), Noggin (Proteintech), B4GalT5 (Sigma), Hsp90 (Santa Cruz Biotechnology), and C/EBPα, and 422/aP2 (obtained from the Department of Biological Chemistry at the Johns Hopkins University School of Medicine).

### Lectin blotting assay

The C3H10T1/2 cells were collected at the indicated time, washed with PBS, scraped off, and collected by centrifugation, and then lysed in RIPA buffer (50 mM Tris-HCl [pH 8.0], 150 mM NaCl,1% NP-40, 0.5% sodium deoxycholate, 0.1% SDS) in the presence of protease inhibitors (Roche) at 4 °C. Cell lysates proteins were separated by SDS–PAGE and transferred onto a PVDF membrane. The PVDF membrane was then incubated in 25 mM H_2_SO_4_ at 4 °C for 1 h to eliminate terminal sialic acid moieties. After being blocked with 5% BSA, the membrane was immunoblotted with biotinylated RCA-I (Vector Laboratories, Burlingame, CA; 1:500 dilutions) for 2 h at room temperature and visualized with horseradish peroxidase-coupled avidin reagent.

### RCA-I lectin pulldown assay

The detection of glycan structures on glycoproteins was achieved by lectin pulldown assays using RCA-I agarose. Total cell lysates in RIPA buffer were incubated with agarose-bound lectins at 4 °C for 16 h. The precipitated proteins were then subjected to western blotting. In RCA-I pulldown assay, to eliminate terminal sialic acid moieties, cells were digested with sialidase for 16 h at 37 °C before collecting.

### Immunofluorescence staining

The C3H10T1/2 cells were fixed with 4% paraformaldehyde for 20 min at room temperature and washed three times with PBS. After blocking and permeabilizing for 1 h with PBS/10% goat serum/0.25% Triton X-100, the cells were incubated with BMPRIA antibody overnight at 4 °C. Cells were washed with PBS and incubated with FITC-labeled second antibody in PBS/5% goat serum for 1 h at room temperature. Finally, the cells were counterstained with DAPI (Vector).

### Flow cytometry

For rupture of cell membranes staining, purified cells were fixed with permeabilization concentrate and diluent (eBioscience) for 30 min at 4 °C. Cells were then washed with Permeabilization Buffer. Cells were incubated with B4GalT5 antibodies for 1 h at 4 °C. Then cells were washed with PBS and incubated with FITC-labeled secondary antibody for 30 min at 4 °C.

For surface staining, purified cells were incubated with BMPRIA antibodies (Abcam and Santa Cruz) for 2 h at 4 °C without being permeabilized. Cells were then washed with PBS and incubated with FITC-labeled secondary antibody for 30 min at room temperature.

For staining macrophages, purified adipose SVF cells were resuspended in PBS/1% BSA (FACS buffer). Macrophage staining was performed for 30 min at 4 °C using the following antibodies: CD45, F4/80, CD11c, CD206 (Bioscience). The cells marked with the antibody were then washed once with FACS buffer before analysis. Flow cytometry analysis was performed using a FACScalibur instrument (BD Bioscience) to measure the fluorescence intensity.

### Adenoviral expression vectors and infection

The adenoviral expression vector pBlock-it (Invitrogen) encoding shRNA of B4GalT5 was constructed according to the manufacturer’s protocols. The shRNA sequences were TGTCACGTACGACGCCTTG. Adenovirus was amplified and purified using Sartorius Adenovirus Purification Kits. Adenovirus solution was injected s.c. adjacent to the inguinal fat pad twice a week for 6 weeks in mice beginning at age 6 week. LacZ shRNA was as a negative control.

### Statistical analysis

Results were expressed as means ± standard deviation (SD). Comparisons between groups were examined by Student’s *t*-test (two-tailed). For comparison of more than two groups with comparable variances, one-way ANOVA and Bonferroni’s post hoc tests were carried out using commercially available software (GraphPad). *P* < 0.05 was considered statistically significant. All experiments were repeated a minimum of three times with triplicate samples and representative data are shown.

## Electronic supplementary material

supplemental figure
