# Supplementary material for: Downregulation of β1,4-galactosyltransferase 5 improves insulin resistance by promoting adipocyte commitment and reducing inflammation
Source: Cell Death Dis. 2018 Feb 7;9(2):196. doi: 10.1038/s41419-017-0239-5 (PMC5833706; doi:10.1038/s41419-017-0239-5)
Supplement: Supplementary file 1 — supplemental figure [file 41419_2017_239_MOESM1_ESM.pdf]

Supplementary figure

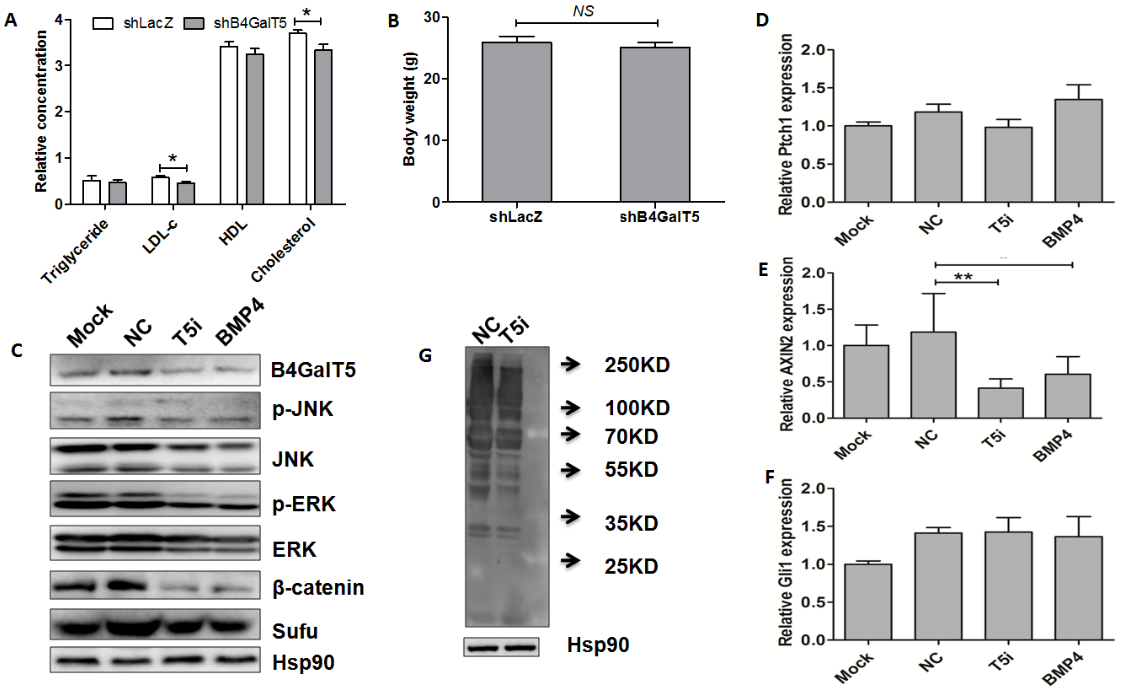

(A)Blood triglycerides, LDL, HDL and cholesterol levels were measured in mice treated with B4GalT5 or LacZ shRNA adenovirus (n=8). (B) Comparison of body weight in HFD mice treated with B4GalT5 or LacZ shRNA adenovirus. (C) Western blot analysis of the different signaling pathways on day 0 using the antibodies indicated above, with Hsp90 as a loading control. (D-F) Expression of Ptch1, AXIN2 and Gli1 in C3H10T1/2 cells with B4GalT5 knockdown on day 0. (G) On day 0, proteins were separated by SDS–PAGE and the binding to RCA-I lectin were analyzed by RCA-I lectin blotting. Hsp90 was served as a loading control.
